# Decoding and modifying dynamic attentional bias in gaming disorder

**DOI:** 10.1098/rstb.2023.0090

**Published:** 2024-10-21

**Authors:** Taiki Oka, Takatomi Kubo, Nao Kobayashi, Misa Murakami, Toshinori Chiba, Aurelio Cortese

**Affiliations:** ^1^ Department of Decoded Neurofeedback, Computational Neuroscience Laboratories, Advanced Telecommunications Research Institute International, Kyoto, Japan; ^2^ Department of Neuropsychiatry, Faculty of Life Sciences, Kumamoto University, Kumamoto, Japan; ^3^ Clinical Psychology, Graduate School of Human Sciences, Osaka University, Suita, Japan; ^4^ Graduate School of Science and Technology, Nara Institute of Science and Technology, Nara, Japan; ^5^ Healthcare Medical Group, Life Science Laboratories, KDDI Research, Inc., Saitama, Japan; ^6^ Department of Psychiatry, Self-Defense Forces Hanshin Hospital, Kawanishi, Japan

**Keywords:** gaming disorder, attentional bias, insula, multivariate pattern analysis, decoded neurofeedback, attentional bias modification

## Abstract

With the spread of smartphones and computer games, concerns have escalated regarding the rising prevalence of gaming disorder. Patients often display attentional biases, unconsciously turning their attention towards gaming-related stimuli. However, attempts to discover and ameliorate these attentional deficits have yielded inconsistent outcomes, potentially due to the dynamic nature of attentional bias. This study investigated neural mechanisms underlying attentional bias state by combining neuroimaging (functional magnetic resonance imaging -fMRI) with an approach-avoidance task tailored to an individual’s gaming preference. We conducted a multivariate pattern analysis of endogenous brain activity in 21 participants with probable gaming disorder. Our analyses revealed that activity patterns in the insula tracked temporal attentional bias states specific to gaming stimuli. A broad network of frontal and parietal regions instead appeared to predict a general temporal attentional bias state. Finally, we conducted a proof-of-concept study for ‘just-in-time’ attentional bias training through fMRI-decoded neurofeedback of insula activity patterns, named decoded attentional bias training (DecABT). Our preliminary results suggest that DecABT may help to decrease the attractiveness of gaming stimuli via a insula- and precuneus-based neural mechanism. This work provides new evidence for the insula as an endogenous regulator of attentional bias states in gaming disorder and a starting point to develop novel, individualized therapeutic approaches to treat addiction.

This article is part of the theme issue ‘Neurofeedback: new territories and neurocognitive mechanisms of endogenous neuromodulation’.

## 1. Introduction

Video and online games provide entertainment for millions of people. However, maladaptive engagement can lead to functional impairments, a phenomenon called ‘gaming disorder’ [1]. As a consequence, gaming disorder has become problematic in today’s technologically advanced society [2] and has been included as an official diagnosis in the International Classification of Diseases 11th Revision [3]. Although recent reviews indicate a prevalence of around 3% [1], which increased during the COVID-19 pandemic [4], effective treatments are still underdeveloped [1].

A common problem with such addictions is attentional bias. Game players often find playing online games satisfying, providing relief from negative moods [5], which over time develops into an attentional bias towards game-related stimuli [6]. This unconscious bias towards addiction-related stimuli is a central factor for symptom prognosis [7,8]. Furthermore, attentional bias has been proposed as a theranostic biomarker that can be modified via attentional bias modification (ABM) [9]. In short, ABM helps reduce bias towards a stimulus by repeatedly encouraging participants to avoid addiction-related stimuli, e.g. gaming, alcohol and drugs, etc., which helps to ameliorate symptoms [10,11].

Although attentional bias towards addictive stimuli has been considered an essential mechanism of gaming disorder, previous reports on the direction of the bias, i.e. towards or away from relevant stimuli, have been inconsistent [12,13]. Moreover, although attempts at ABM were reported as effective, the effects varied among studies [14,15]. One reason for such variability in study outcomes may be that attentional bias is unstable within each individual, fluctuating in time [16,17]. Recent research suggested that traditional assumptions should be revisited and that therapeutic measures should consider attentional bias as a dynamic process [18]. Such temporal changes in attentional bias may have affected previous results, and interventions on attentional bias should consider this fluctuation [13,19]. Furthermore, attentional bias towards addiction-related stimuli is only found in the presence of high craving, which is closely related to symptom onset [20]. Such attentional fluctuation is thought to be related to endogenous brain activity [21,22]. Although temporal variation in attentional bias could be related to brain states, its neural basis has yet to be well investigated, despite its clinical importance.

Here, we first attempted to clarify which brain regions are important for attentional bias states in gaming disorder, using an approach–avoidance task that measures implicit biases. We focused our analysis on a key set of addiction disorder-related brain regions, especially those associated with attentional bias, i.e. the ventral medial prefrontal cortex (vmPFC), insula, amygdala, hippocampus, ventral striatum (VS), mesolimbic region and cerebellum [23–25]. We used multivariate pattern analysis (MVPA) to classify patterns of voxels representing the attentional state (biased and unbiased). Our analysis targeted pre-stimulus brain activity to consider endogenous neural fluctuation [26,27]. We selected MVPA because previous functional magnetic resonance imaging (fMRI) studies on attentional bias have yielded inconsistent findings [27]. Indeed, complex dynamical spatial patterns of brain activity, which are not well addressed by conventional univariate analysis, may represent attentional bias. Our MVPA results suggest that gaming-related temporal attentional bias involves a constellation of brain regions implicated in attentional switching and action preparation, with the insula playing a prominent role [28]. A control experiment based on general gaming stimuli, instead of individualized ones, reinforced the specificity of this gaming addiction-related attention effect in the insular cortex.

If neural mechanisms can be identified, revealed neural foundations could be next-generation therapeutic targets [29]. In particular, decoded neurofeedback (DecNef) is a method of intervening in behaviour based on real-time brain activity [30]. While standard neurofeedback can only manipulate macro units, such as the average activity level of a specific brain region, and the region to be manipulated must be determined by prior knowledge, DecNef allows interventions to be implemented using brain activity patterns that represent specific behavioural and cognitive states [31]. As mentioned, because attentional bias fluctuates in response to transitions in intrinsic neural activity, just-in-time interventions that capture the target state should enhance the effect [32]. Applying DecNef makes it possible to present the intervention when the neural attentional bias is maximal [33]. In addition, while a conventional ABM task is a series of simple behavioural trials that could evoke boredom, a gamification approach in which the timing is unreadable by the participant could help to keep motivation and enhance their engagement [34].

Here, we show preliminary results from a proof-of-concept approach that combines ABM with real-time fMRI decoding of brain activity: decoded attentional bias training (DecABT). DecABT captures the attentional ‘peak’ of a participant based on the brain decoder, which can produce stimuli to modify participants’ attention in a just-in-time manner. As the brain region to construct the brain decoder for the training, we chose the insula because (i) it is associated with spontaneous craving in addiction [35] and (ii) it reflects attention to game stimuli most sensitively in our region of interest (ROI)-based MVPA. To evaluate the training’s efficacy, we analysed not only subjective and behavioural indexes but also brain functional changes to examine whether DecABT could affect the function of the insular cortex or closely related brain regions. In line with previous work, DecABT effects may be detectable as changes in brain function, even when not apparent at the subjective or behavioural levels [36]. Our results suggest that DecABT can change brain activity related to cue reactivity and reward processing in addiction. Optimizing closed-loop attentional intervention, by considering neural fluctuation, may ameliorate underlying cognitive deficits and facilitate treating addiction.

## 2. Research design and methods

### (a) Participants

Twenty-three individuals (six females) with probable gaming disorder were recruited through advertisements in local universities and through an online research company (Macromill, Inc., https://monitor.macromill.com/). Inclusion criteria were as follows: (i) a score ≥3 points on the Internet Gaming Disorder Scale (IGDS-J) [37,38] in screening. A total score ≥5 is defined as full syndromal gaming disorder based on diagnostic criteria of the DSM-5 [38] and ≥3 is defined as subsyndromal gaming disorder [39]. Therefore, in this study, a participant with a total score of IGDS ≥3 was defined as having probable gaming disorder (details of the questionnaire are shown in electronic supplementary material, methods). (ii) Age between 18 and 60 years old; (iii) fluent Japanese speakers; and (iv) right-handed. Exclusion criteria were as follows: (i) visual dysfunction that could not be corrected or adjusted and would prevent one from recognizing images on a monitor; (ii) positive diagnoses of current or recent psychiatric disorders; (iii) any history of head trauma, presence of metal in the body or other contraindications to fMRI scanning; (iv) with excessive head-motion during fMRI scanning; (v) task accuracy lower than 80%; and (vi) troubles that may distort data, e.g. sleep during the task in the scanner. We excluded two participants from the analysis: one’s accuracy was lower than 80%, and one fell asleep in the scanner. Finally, we analysed 21 participants (mean age 29.0 yr; s.e.m. ± 2.2; six females). Based on each individual availability and target decoder classification accuracy, seven participants (three females) engaged in the proof-of-concept DecABT (average decoding accuracy: 63.5 ± 2.7%; see electronic supplementary material, table S1 for individual decoding accuracies).

Seven of the 21 participants from the main task and six additional participants took part in a control experiment (see §2c). Of the six additional participants, one met the criteria for probable gaming disorder. The control experiment thus included eight participants with probable gaming disorder and five healthy participants.

### (b) Task materials

**Figure 1.**
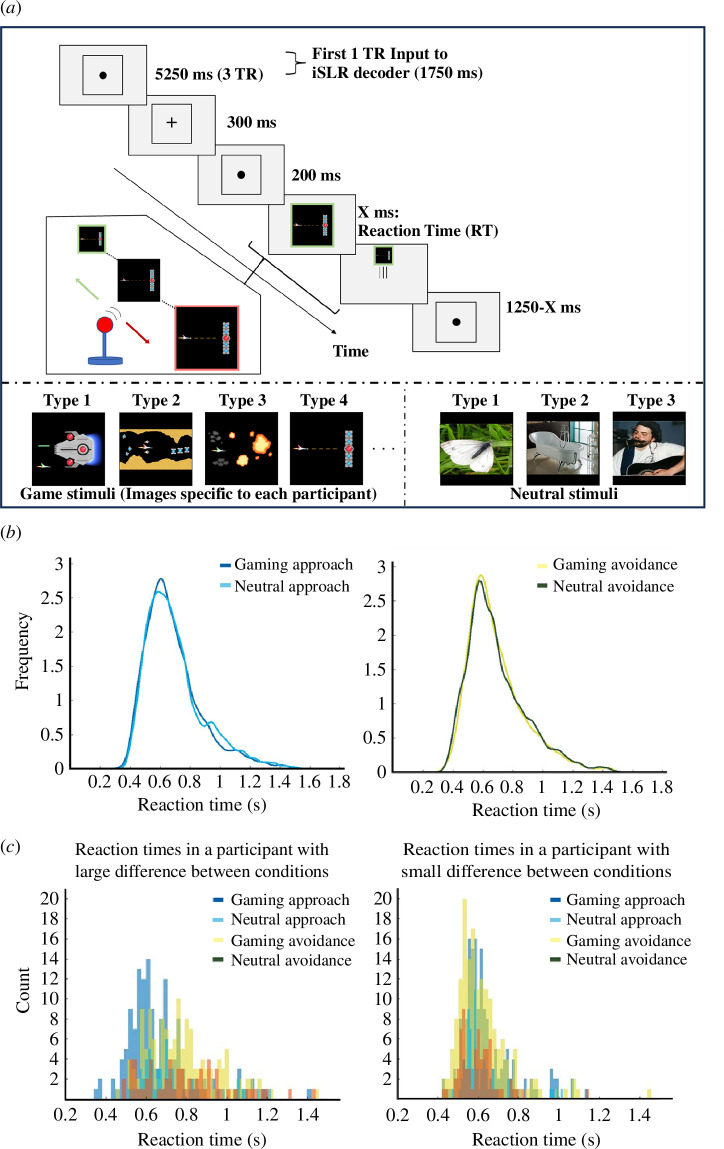
Experimental paradigm and behavioural results. (*a*) Overview of the approach–avoidance task procedure. In each trial, participants had to pull the joystick towards themselves, i.e. approach, if the frame colour of the stimulus was red, or push the joystick away, i.e. avoid, if the frame colour was green. They were instructed to respond as quickly as possible when the stimulus appeared. Each block contained imbalanced stimuli (56 gaming and 24 neutral trials) to collect sufficient responses in gaming stimuli for decoding. (*b*) Reaction time distributions in each direction (approach/avoidance). Figures were drawn using kernel-fitted distribution (bandwidth: 0.025) for each direction (approach/avoidance). Individual reaction time data were pooled across participants within each condition. (*c*) Reaction time distributions from two example participants. Histograms of reaction time differences between conditions (gaming/neutral approach and gaming/neutral avoidance) from an example participant with a large difference and one with a small difference. In both (*b*) and (*c*), approach trials of gaming and neutral stimuli are coloured blue and cyan, avoidance trials of gaming and neutral stimuli are coloured yellow and dark green.

The gaming condition used game images prepared from the two game titles most played by each participant and were collected from the internet. The neutral condition instead used neutral stimuli obtained from two image databases unrelated to gaming [40,41] (for stimuli examples, see figure 1*a*). In the control experiment, game stimuli were ‘general’ stimuli (e.g. Dungeons & Dragons) common to all participants and collected from the internet. The stimuli were displayed on a screen inside the MRI scanner using a projector (DLA-HD10KHK) and a mirror system. Participants responded to the stimuli using an MRI-compatible joystick (HHSC-JOY-5; Current Designs, Inc., PA, USA).

### (c) Task to measure attentional bias

We assessed the attentional bias of each participant using an approach–avoidance task (figure 1*a*). Participants pushed or pulled an MRI-compatible joystick in response to the frame colour of the cue (red or green). This task can assess implicit approach– avoidance bias tendencies related to addictive stimuli with a high degree of ecological validity based on movement towards or away from the images [42]. Trials were presented in at most six blocks (average: 5.6, s.e.m. = 0.2). Each block started with a baseline period (21 s), followed by a pseudo-randomized sequence of 56 gaming and 24 neutral trials. This imbalanced ratio was designed to collect sufficient responses in gaming stimuli to decode the attentional bias state. The task featured a zooming function that increased/decreased the cue size as participants moved the joystick towards/away to maximize the similarity between approach and avoidance [43].

### (d) Data acquisition

The Psychophysics Toolbox for Matlab (http://psychtoolbox.org/) was used to conduct the experiments. A 3.0 T scanner (Prisma; Siemens, Erlangen, Germany) with a 64-channel head coil was used to collect fMRI neuroimaging data. We scanned 76 interleaved axial slices that were 2.0 mm thick without gaps, parallel to the anterior–posterior commissure line, using a T2*-weighted gradient-echo multiband echo-planar imaging (MB-EPI) sequence (repetition time (TR) = 1750 ms, echo time (TE) = 30.0 ms, flip angle (FA) = 70°, field of view (FOV) = 200 × 200 mm^2^, resolution = 2 × 2 mm^2^, MB factor = 4, voxel size = 2 × 2 × 2 mm^3^). We obtained 348 volumes for the decoding session and 210 for DecABT for each run. Each run included additional dummy scans at the beginning of scanning for signal stabilization. All individuals underwent a magnetization-prepared rapid acquisition gradient echo technique to acquire high-resolution T1-weighted images of the entire brain for anatomical reference (MPRAGE; TR = 2250 ms, TE = 3.06 ms, FA = 9°, FOV = 256 × 256 mm^2^, voxel size = 1 × 1 × 1 mm^3^). Participants operated a joystick attached inside the MRI scanner with an adhesive sheet, positioned to their right-hand side to ensure natural push and pull movements.

### (e) Data analysis

#### (i) Extracting trial-level attentional bias

**Figure 2.**
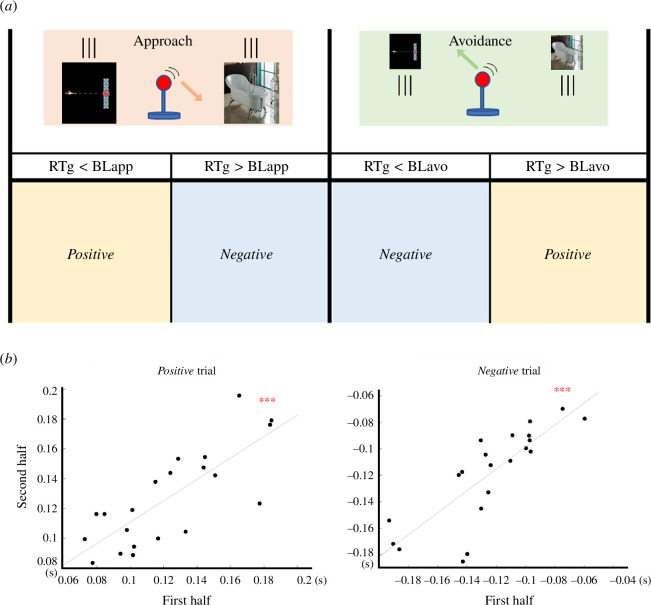
Behavioural labelling for MVPA and within-subject reliability of the trial-level attention bias state. (*a*) Defining trial-level attention bias state (positive/negative). The average reaction time on neutral trials (in approach and avoidance, separately) in each block was used as the baseline (BLapp/avo). If reaction times for trials pushing gaming stimuli (RTg) were slower than the baseline (RTg > BLavo) or reaction times for pulling gaming stimuli were quicker than the baseline (RTg < BLapp), they were labelled as ‘*positive*’, meaning attention was biased towards gaming stimuli. In the opposite case, they were labelled as ‘*negative*’, meaning attention was biased away from gaming stimuli. (*b*) Split-half reliabilities of each index. This graph shows a scatterplot of the correlation between the average of reaction time differences for each label in the first and second halves of trials. Each dot represents the average of each half for a given participant. The line represents the least squares fit. Red asterisks represent *p*_FDR_ (***<0.001).

After removing inappropriate trials (see electronic supplementary material, methods and figure S1a), we used a previously published definition to compute trial-level attentional bias [40,44]. In short, the average reaction time on neutral trials (approach/avoidance, respectively) in each block was used as the approach/avoidance baseline. This baseline is an empirical reference to determine how the reaction time (RT) of each participant on each pulling or pushing gaming trial differed from his/her neutral trials [40]. That is, if RTs for pushing gaming stimuli, i.e. avoidance, were slower than the avoidance baseline, or RTs for trials pulling gaming stimuli, i.e. approach, were quicker than the approach baseline, they were labelled as ‘*positive*’, meaning attentional state towards gaming stimuli. In the opposite case, they were labelled as ‘*negative*’, meaning attentional state away from gaming stimuli (see figure 2*a*). The labelling is based on an existing theory of approach–avoidance behaviour [41] and is supported by several studies that have used the approach–avoidance task (AAT) to investigate attentional biases in various contexts [45,46]. Since the tendency to approach the target of dependence is thought to be related to problematic behaviours and symptoms in addiction science [23], ‘*positive*’ indicates a biased state, while ‘*negative*’ indicates an unbiased state. The split-half, within-participant reliability of the trial-wise attentional bias was high (*positive*: Spearman–Brown *ρ* = 0.88, *p* < 0.001; *negative*: Spearman–Brown *ρ* = 0.90, *p* < 0.001; see figure 2*b*). Moreover, we also calculated differences between neutral approach/avoidance trials from approach/avoidance baselines in a control MVPA analysis (fake trial-level index; see §2*e*(iv) for a detailed explanation).

#### (ii) fMRI pre-processing for multivariate pattern analysis

Blood oxygen level dependence (BOLD) signals in native space were pre-processed in MATLAB (R2020b; MathWorks) using SPM12 and in-house code. All functional images underwent three-dimensional motion correction. No spatial or temporal smoothing was applied. Rigid-body transformations aligned functional images with each participant’s structural image. Region-of-interest (ROI) masks were used to extract relevant voxels. Time courses of BOLD signal intensities from each voxel were shifted by 5.25 s (3 TRs) to account for the hemodynamic delay. We excluded voxels with exceptionally low BOLD signal intensities (mean < 80) or those with considerable variance (s.d. > 8). A linear trend was removed from time courses, which were further *z*-score-normalised for each voxel in each block to minimize baseline differences across blocks. Then, we generate feature vectors by extracting the first TR (1750 ms) of the pre-stimulus period from each trial (see figure 1). We used only the first TR of each trial for decoder construction to extract pre-stimulus brain activity alone, limiting as much as possible the unwanted effect of stimulus presentation and response.

#### (iii) Whole-brain univariate analysis

We conducted a univariate analysis to check the effect of the direction of each gaming-neutral condition in the approach–avoidance task. For pre-processing, images underwent motion correction, reorientation and realignment to the first volume. Subsequently, T1-co-registered volumes were normalised using an MNI (Montreal Neurological Institute) template. Finally, images were smoothed with an isotropic 8 mm FWHM three-dimensional Gaussian filter. A general linear model (GLM) was used to identify the BOLD activation of different event types, for each voxel. Specifically, four regressors were generated for gaming/neutral approach and gaming/neutral avoidance events. Onset was defined as the timing of each stimulus presentation, and duration was based on the reaction time. Note that for this analysis, we extracted the stimulus onset and treated the reaction time of each trial as durations, which differed from the MVPA. Additional parameters were incorporated as covariates of no interest, including fixation, mean white matter and cerebrospinal fluid signal, global signal, six motion parameters and framewise displacement. Then, we ran two group-level analyses. The first contrasted gaming approach > neutral approach, and the second contrasted gaming avoidance > neutral avoidance. Avoidance conditions (push gaming/neutral) were considered of no interest and regressed out in the comparison of approach condition (pull gaming/neutral) GLM and vice versa for the comparison of avoidance condition GLM. Additionally, to check the effects of stimuli and direction themselves, we constructed two contrasts (game versus neutral and approach versus avoidance).

#### (iv) Region of interest-based multivariate pattern analysis

Our primary ROI-based analysis targeted the vmPFC, insula, amygdala, hippocampus, VS, mesolimbic region (combined substantia nigra (SN)/ventral tegmentum area (VTA)/VS) and the cerebellum. These ROIs were chosen based on previous studies [47–50] concerning attentional bias in addiction and dopaminergic function, which is also associated with addiction [51]. We defined the bilateral amygdala, hippocampus, cerebellum and insula through the Automated Anatomical Labelling 3 atlas (AAL3) [52], accessible via the Wake Forest University PickAtlas toolbox for SPM12. The VS was defined using a 12 mm radius, centred at *x* = ± 12, *y* = 10, *z* = −6 [53]. The vmPFC was defined using an 8 mm radius, centred at *x* = 0, *y* = 46, *z* = −7 [54]. The mesolimbic ROI was defined as the combination of three regions as follows: SN with a 3 mm radius at coordinates *x* = −10.1, *y* = −18.9, *z* = −11.6 and *x* = 11.3, *y* = −18.7, *z* = −11.7 [55]; VTA with a 3 mm radius at coordinates *x* = −2.7, *y* = −15.9, *z* = −13.9 [55] and *x* = 4.1, *y* = −15.9, *z* = −13.9; and VS as given above.

As endogenous brain activity for decoding, we used the first TR (duration 1.75 s) fMRI signals within the inter-trial interval immediately before stimulus presentation. We used iterative sparse logistic regression (iSLR) [56] to construct individual binary classifiers (positive/negative). iSLR automatically selects relevant voxels in ROIs for MVPA with optimization using an iterative approach. That is, selected voxels from pattern vectors are eliminated at each iteration, and only features with unassigned weights are used for the subsequent iteration. Each test sample label is calculated as the multiplied probability across iterations, before entering a logit function to obtain binary outputs. The iSLR was run over 10 iterations. Decoding accuracy was computed using leave-one-run-out cross-validation, averaged over all cross-validation folds. Because there were different numbers of trials in each class, we performed a simple bootstrap balancing procedure (see electronic supplementary material, figure S1b). In each fold, the majority class was randomly downsampled 10 times to match the size of the minority class, and iSLR was performed at each resampling. Final decoding accuracy for a given participant and ROI was taken as the average of 10 resampling runs. Furthermore, we conducted three types of control MVPAs: training in gaming and testing in neutral indexes, i.e. fake trial-level index defined as differences from each RT of neutral trial and baseline calculated by average neutral trials within a block (gaming→neutral condition); training in neutral and tested in neutral indexes (neutral condition); and training in neutral and tested in gaming indexes (neutral→gaming condition). We also conducted the same analyses using data from the control experiment based on general gaming stimuli. Moreover, to check if the decoding performances might depend on particular trial types (i.e. approach/avoidance), we further computed decoding accuracy in the held-out data separately for trials belonging to approach and avoidance.

#### (v) Search-‘region of interest’ multivariate pattern analysis

Considering that the ROI analysis may have been too narrowly defined or that hemispheric asymmetries existed, we conducted an exploratory search-ROI analysis over the whole brain [57]. For this purpose, we used a standard parcellation based on the AAL3 [52] and selected all 166 regions of this brain atlas. We applied the same decoding process based on the gaming condition with the above ROI-based analysis for each AAL3 region. The same process was done in gaming and neural conditions to examine the difference between game and neutral conditions.

#### (vi) Statistical analysis

For behavioural analysis, we tested RT differences between conditions: game versus neutral, game approach versus neutral approach and game avoidance versus neutral avoidance using *t*-tests with *p* < 0.05 corrected for the false discovery rate (FDR) [50] after logarithmic transformation of individual RT data. Furthermore, we tested the difference between trial-level bias (*positive/negative*) of gaming and neutral trials using *t*-tests with *p*_FDR_ < 0.05.

In whole-brain univariate analysis, group-level activity maps were created using one-sample *t*-tests, thresholded at *p*_unc_ = 0.001 at the peak voxel level and cluster size at *p*_FDR_ < 0.05. In ROI-based MVPA, two-sided *t-*tests evaluated decoding accuracies against the theoretical chance level (50%) or in direct comparison to other control MVPA results with *p*_FDR_ < 0.05 across all ROIs and four conditions (gaming, gaming→neutral, neutral and neutral→gaming). For the exploratory search-ROI MVPA, a *t*‐test evaluated whether the decoding accuracy of each ROI was significantly higher than the theoretical chance level (50%) with a strict threshold for exploration (*p*_FDR_ < 0.001).

### (f) Decoded attentional bias training

#### (i) Overall procedure

**Figure 3.**
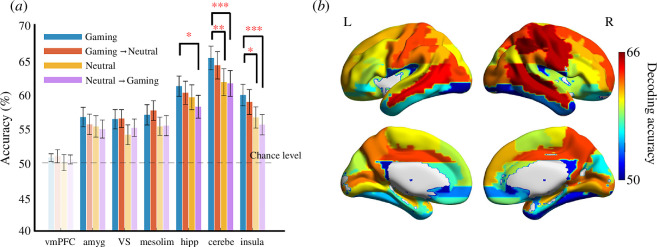
Results of MVPA. (*a*) Classification accuracy in each pre-selected ROI. Results of decoding accuracy in each ROI, which represent how each brain region classified the attentional bias state (positive/negative). The colour intensity of each bar reflects its significance compared to the theoretical chance level (50%; full opacity: *p*_FDR_ < 0.001, mid-opacity: *p*_FDR_ < 0.05, high transparency: not significant). Red asterisks represent *p*_FDR_ (* < 0.05, ** < 0.01, *** < 0.001) in comparisons across conditions. vmPFC, ventral medial prefrontal cortex; amyg, amygdala; VS, ventral striatum; mesolim, mesolibic region including substantia nigra, ventral tegmental area and VS; hipp, hippocampus; cerebe,cerebellum; insula, insula. (*b*) Brain map of a group-level search-ROI analysis. The colour bar represents decoding accuracy (%). Warm colours indicate better prediction, i.e. red–orange regions, whereas colder colours indicate prediction close to chance level (blue regions). Table 1 displays all significant results. Visualization based on BrainNet viewer [58].

Once their targeted decoder was constructed from the decoding session (approach–avoidance task), selected participants completed DecABT training over three consecutive days (figure 3). The first and last days included two blocks with approach–avoidance tasks to assess their attentional bias, a resting-state scan, and up to five blocks of DecABT (first day: 3.4 ± 0.4, last day: 3.4 ± 0.3). Specifically, resting-state fMRI scanning, two blocks of AAT and training were performed in order on day 1 and in reverse order on day 3. Additionally, participants answered a Problematic Online Gaming Questionnaire (POGQ) [59] to assess the change in gaming disorder severity from pre- to post-training (details of the questionnaire are shown in electronic supplementary material, methods). In the approach–avoidance task, participants performed 80 trials (40 gaming/40 neutral images) over two blocks. During the second day, participants completed up to nine blocks (8.4 ± 0.4) of DecABT to modify approach bias to gaming stimuli. For this paradigm, we used the decoder constructed from activity patterns in the insula, representing *positive* versus *negative* attentional states towards gaming stimuli. We chose the insula because (i) that region is associated with spontaneous craving in addiction [60] and (ii) it reflected the most specific attention effect to game stimuli in the ROI-based analysis (as opposed to other regions, which showed similar-sized non-specific effects to neutral stimuli, see §3). Importantly, we used the whole dataset to train the decoder in the insula used for the DecABT experiment.

**Table 1.** Results of the whole-brain search-ROI analysis. Results include regions significant at *p*_FDR_ < 0.001. Labels of each region are based on AAL3 [52].

region	statistical values				region	statistical values			
	accuracy	s.e.m.	*t* stat	Cohen’s *d*		accuracy	s.e.m.	*t* stat	Cohen’s *d*
Precentral_L	65.12	1.66	9.112	2.148	Fusiform_R	55.11	0.98	5.231	1.233
Precentral_R	62.02	1.80	6.668	1.572	Parietal_Inf_L	65.58	1.67	9.326	2.198
Frontal_Sup_2_L	62.53	1.74	7.198	1.697	Parietal_Inf_R	62.52	1.81	6.914	1.630
Frontal_Sup_2_R	60.90	1.98	5.502	1.297	SupraMarginal_L	61.86	1.77	6.697	1.578
Frontal_Mid_2_L	61.49	1.69	6.777	1.597	SupraMarginal_R	62.27	1.86	6.604	1.557
Frontal_Mid_2_R	59.67	1.87	5.176	1.220	Angular_L	59.67	1.44	6.716	1.583
Frontal_Inf_Oper_L	58.85	1.46	6.062	1.429	Angular_R	60.93	1.96	5.567	1.312
Frontal_Inf_Oper_R	60.19	1.47	6.917	1.630	Precuneus_L	62.93	1.43	9.048	2.133
Frontal_Inf_Tri_L	60.41	1.86	5.587	1.317	Precuneus_R	62.37	1.87	6.629	1.562
Frontal_Inf_Tri_R	58.51	1.57	5.405	1.274	Paracentral_Lobule_L	59.60	1.76	5.464	1.288
Frontal_Inf_Orb_2_L	56.73	1.29	5.205	1.227	Paracentral_Lobule_R	60.36	1.56	6.661	1.570
Supp_Motor_Area_L	59.03	1.70	5.312	1.252	Pallidum_R	54.81	1.11	4.324	1.019
Supp_Motor_Area_R	59.85	1.70	5.796	1.366	Temporal_Sup_L	59.41	1.54	6.117	1.442
Frontal_Sup_Medial_L	60.52	1.72	6.104	1.439	Temporal_Sup_R	59.83	1.55	6.356	1.498
Frontal_Sup_Medial_R	59.46	1.81	5.228	1.232	Temporal_Pole_Sup_L	61.64	1.65	7.062	1.664
Rectus_L	58.19	1.57	5.226	1.232	Temporal_Pole_Sup_R	60.58	1.78	5.954	1.403
Rectus_R	57.45	1.54	4.823	1.137	Temporal_Mid_L	64.94	1.44	10.386	2.448
OFCmed_R	59.00	1.12	8.001	1.886	Temporal_Mid_R	64.14	1.79	7.907	1.864
OFCant_L	56.42	1.46	4.402	1.038	Temporal_Inf_R	55.71	1.13	5.070	1.195
OFCpost_L	58.32	1.57	5.291	1.247	Cerebellum_Crus1_L	61.64	1.37	8.490	2.001
OFCpost_R	60.50	1.57	6.697	1.579	Cerebellum_Crus1_R	59.57	1.23	7.777	1.833
OFClat_L	56.77	1.32	5.143	1.212	Cerebellum_Crus2_L	62.45	1.58	7.860	1.853
Insula_L	58.55	1.61	5.310	1.252	Cerebellum_Crus2_R	63.57	1.84	7.370	1.737
Insula_R	59.29	1.79	5.200	1.226	Cerebellum_4_5_L	57.34	1.46	5.025	1.185
Cingulate_Mid_L	62.74	1.73	7.348	1.732	Cerebellum_6_L	53.54	0.73	4.839	1.141
Cingulate_Mid_R	62.72	1.86	6.843	1.613	Cerebellum_7b_L	62.68	1.61	7.870	1.855
Hippocampus_L	60.84	1.36	7.963	1.877	Cerebellum_7b_R	58.84	1.64	5.402	1.273
Hippocampus_R	60.86	1.79	6.061	1.429	Cerebellum_8_L	62.18	1.73	7.023	1.655
ParaHippocampal_L	55.86	1.36	4.309	1.016	Cerebellum_8_R	61.05	1.70	6.484	1.528
Amygdala_R	57.49	1.57	4.758	1.122	Cerebellum_9_L	58.59	1.80	4.770	1.124
Calcarine_L	61.98	1.56	7.662	1.806	Cerebellum_9_R	59.69	1.47	6.577	1.550
Calcarine_R	62.47	1.51	8.274	1.950	Cerebellum_10_L	58.91	1.65	5.401	1.273
Cuneus_L	55.66	1.11	5.097	1.202	Cerebellum_10_R	58.26	1.31	6.309	1.487
Cuneus_R	59.02	1.33	6.794	1.602	Vermis_4_5	60.86	1.66	6.540	1.541
Lingual_L	60.68	1.51	7.059	1.664	Vermis_8	59.90	1.23	8.033	1.893
Lingual_R	60.43	1.63	6.413	1.512	Vermis_9	55.26	0.95	5.565	1.312
Occipital_Sup_L	59.24	1.50	6.146	1.449	Thal_LGN_R	55.11	1.10	4.625	1.090
Occipital_Sup_R	59.70	1.47	6.618	1.560	Thal_MGN_R	54.21	0.87	4.847	1.142
Occipital_Mid_L	62.33	1.56	7.898	1.862	Thal_PuI_R	54.02	0.88	4.582	1.080
Occipital_Mid_R	62.94	1.71	7.573	1.785	Thal_PuM_L	53.47	0.78	4.464	1.052
Postcentral_L	64.79	1.70	8.696	2.050	ACC_pre_L	57.02	1.51	4.636	1.093
Postcentral_R	62.81	1.78	7.190	1.695	ACC_pre_R	56.75	1.53	4.419	1.042
Parietal_Sup_L	63.32	1.68	7.927	1.869	ACC_sup_L	58.56	1.80	4.768	1.124
Parietal_Sup_R	60.86	1.78	6.101	1.438	ACC_sup_R	57.16	1.61	4.447	1.048

#### (ii) Attention bias modification through decoded fMRI neurofeedback

The basic instruction was the same as for the main approach–avoidance task. Participants pushed or pulled an MRI-compatible joystick in response to the frame colour of a cue presented on the monitor. To avoid stimulus habituation, the task featured different but equivalent cues, i.e. different pictures from the same game, used in the neurofeedback and approach–avoidance task. First, participants were instructed to passively look at a fixation circle. During this fixation, the online decoder calculated the probability of an attentional bias state from the multivoxel pattern of fMRI activity in the insula on each TR. The higher the probability, the more the brain activity showed an implicit attentional bias state towards gaming. A stimulus appeared when the probability was above 95% in order to provide intervention at the optimal time when the attentional bias state towards gaming pictures was high. If the probability did not exceed the threshold within a fixed time window (15 TR), the stimulus did not appear in the trial (NG trial; average of 1.2 times ± 0.2 within one block). Following a protocol reported previously [61], gaming pictures were consistently associated with avoidance in our experiment, while neutral ones were consistently associated with approach. Thus, participants were effectively trained to avoid gaming pictures based on their brain states. When the insula neural decoder detected a biased state, stimuli were presented. These stimuli could be either gaming or neutral, with a higher chance of gaming. Game images had a probability of appearance of 69.1 ± 0.2%. Importantly, participants were not informed about the underlying rule determining the timing and nature of stimuli. Further details of the paradigm and materials for this feasibility study are described in electronic supplementary material, DecABT methods and figure S2. We analysed the differences in self-reported symptom severity of gaming disorder and behavioural indexes from pre- to post-training. In addition, we analysed functional brain activation data during pre- and post-AAT to check whether neuroplastic changes might have further underlied DecABT training.

## 3. Results

### (a) Behavioural results

Reaction time distributions in the approach–avoidance task are shown in figure 1 (see electronic supplementary material, figures S3 and S4 for individual participants’ results). At the group level, there were only small or no differences between overall gaming and neutral stimuli (*t*(8049) = −1.25, *p*_FDR_ = 0.26, *dz* = 0.09), gaming approach and neutral approach (*t*(4012) = −2.31, *p*_FDR_ = 0.05, *dz* = −0.07, i.e. neutral trials were slower than gaming trials), gaming avoidance and neutral avoidance (*t*(4035) = 0.49, *p*_FDR_ = 0.62, *dz* = 0.02) and a difference in trial-level ‘positive’ attentional bias states between gaming and fake index from neutral stimuli (*t*(5140) = 1.29, *p*_FDR_ = 0.26, *dz* = 0.04). There was, however, a significant difference in trial-level ‘negative’ attentional bias states between gaming and fake index from neutral stimuli, which may indicate less away from gaming stimuli compared to neutral stimuli when considering the subtraction from the baseline (*t*(3593) = −8.38, *p*_FDR_ < 0.001, *dz* = 0.32).

### (b) Univariate analyses

The contrast gaming approach > neutral approach showed a significant cluster of activity centred in the right angular gyrus (*p*_FDR_ = 0.026, *z* = 3.83, MNI coordinates: (28 −56 46), cluster size: 218) and in the left pregenual anterior cingulate cortex (*p*_FDR_ = 0.026, *z* = 3.53, MNI coordinates: (−6 44 −4), cluster size: 191). Instead, the contrast gaming avoidance > neutral avoidance showed a significant cluster centred in the right lingual gyrus (*p*_FDR_ = 0.040, *z* = 4.09, MNI coordinates: (8 −38 −6), cluster size: 156; see details in electronic supplementary material, table S2 and figure S5). These results are consistent with previous fMRI studies on attentional bias, such as biased attention towards foods in obesity and craving in gaming disorder [62,63]. The results of gaming versus neural and approach versus avoidance are shown in electronic supplementary material, table S2 and figure S5.

### (c) Multivariate pattern analysis

#### (i) Region of interest-based multivariate pattern analysis results

Average decoding accuracies for classifying positive versus negative attentional bias states in the gaming condition were significantly higher than chance in the mesolimbic region, hippocampus, cerebellum and insula. However, results of control analyses (in which other conditions were used to predict gaming classes or vice versa) also showed significant decodability from activity patterns in the mesolimbic region, hippocampus and cerebellum (figure 3*a*). These results indicate that the decoder from the mesolimbic region, hippocampus and cerebellum may not be able to separate gaming-related bias and simple response variability. The hippocampus, cerebellum and insula showed a significant difference in the gaming condition versus the cross-decoding condition (in which the model learned from neutral trials and was tested in gaming trials; hippocampus: *t*(20) = 3.92, *p*_FDR_ = 0.002, *dz* = 0.86; cerebellum: *t*(20) = 5.28, *p*_FDR_ < 0.001, *dz* = 1.15; insula: *t*(20) = 4.22, *p*_FDR_ = 0.001, *dz* = 0.92). However, only the cerebellum and the insula showed an additional significant difference in gaming versus neutral conditions (in which the model was trained and tested on trials in the same condition cerebellum: *t*(20) = 3.29, *p*_FDR_ = 0.007, *dz* = 0.71; insula: *t(*20) = 2.55, *p*_FDR_ = 0.033, *dz* = 0.56). We also tested whether there was a difference in decoding performance by direction (approach/avoidance) in each decoding condition. There were significantly higher accuracies in avoidance in some ROIs/decoding types. In the gaming-only decoding condition, there were significant differences in the amygdala, VS and mesolimbic region. In the neutral-only condition, there was a significant difference in the hippocampus. In the cross-decoding condition, the model learned from neutral trials and was tested in gaming trials, there were also significant differences in the hippocampus and insula (see electronic supplementary material, figure S6). There was no significant difference in all conditions from the control experiment (see electronic supplementary material, figure S7). A control analysis did not reproduce these results, i.e. control experiment with general gaming stimuli instead of participant-specific gaming stimuli (electronic supplementary material, figure S8a), which indicates that insular activity reflects a personalized gaming-specific effect. Moreover, control experiment results were consistent in subgroup analyses separating healthy participants and those with gaming disorder (electronic supplementary material, figure S8b,c). However, since the sample size of the control experiment (*n* = 13) was smaller than the main experiments (*n* = 21), we performed bootstrap resampling (1 00 000 times) to extract *n* = 13 from 21 participants in the main experiment and test whether the main experiment results would hold even after balancing the sample size. The results showed significant differences between the main and control results in the insula (*p* = 0.03) but not in the cerebellum (*p* = 0.85; also see electronic supplementary material, figure S9), indicating the effect was robust to sample size variation. To further validate that the insula effect is specific to personalized gaming stimuli in probable gaming disorder, we also tested the interaction between condition (gaming versus neutral) and experiment (main versus control) on the insula decoding accuracy. We used a linear mixed-effect model with a random intercept (‘accuracy ∼ condition * experiment + (1|participants)’, in Wilkinson notation). While the effect was in the expected direction, the interaction was not significant (*p* = 0.11).

#### (ii) Search-‘region of interest’ multivariate pattern analysis results

As in the standard ROI-based MVPA, this exploratory search-ROI analysis focused on classifying *positive* versus *negative* attentional bias state in the gaming condition (in which the model learned from gaming trials and was tested in gaming trials). We found broad regions, including several fronto-parietal and temporal areas, to be predictive of attentional bias state. Search-ROI analysis results were consistent with selected *a priori* ROI MVPA results. Other regions whose accuracies were equal to or higher than the insular cortex in the selected ROI-based analysis were in the frontal, occipital and parietal lobes. In particular, decoding accuracies based on the left precentral gyrus and the left inferior parietal gyrus were the highest (>60%). These are regions related to attentional bias in gaming disorder [64]. Decoding accuracies from these regions were higher than those from the control analysis using trials with neutral stimuli. However, there were no significant results after adjusting for multiple comparisons (FDR; see electronic supplementary material, figure S10 and table S3).

### (d) Decoded attentional bias modification: preliminary results

**Figure 4.**
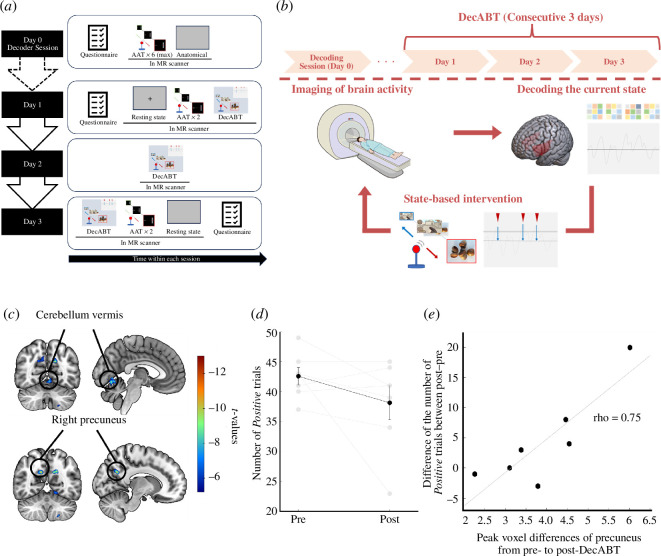
Overview of the experimental procedure, DecABT and its results. (*a*) Experimental procedure. On day 0, participants conducted six runs of AAT in an MRI scanner for the decoder construction. The dotted line arrows connecting day 0 to day 1 indicate the variable time between the two sessions (average 5 days; s.e.m. = 0.7). On day 1, after participants answered a self-reported POGQ, they took part in a resting-state scan, two runs of AAT and DecABT. On day 2, they only took part in DecABT. Day 3 was conducted in the reverse order of day 1. As indicated by the arrow at the bottom, each day’s experiments were conducted in order from left to right. Anatomical, T1-weighted structural MRI; AAT, approach–avoidance task; DecABT, decoded attentional bias training. (*b*) Scheme of DecABT. First, participants lie in the scanner and see a fixation cross. During this period, the brain decoder calculates the neural attentional state of participants. The algorithm produces a stimulus if the decoder captures an attentional ‘peak’ (high probability of biased attention state). This procedure was designed to modify participants’ attention to avoid addiction-related stimuli or approach neutral stimuli in a just-in-time manner. (*c*) Results from whole-brain voxel-wise GLM analyses reveal the difference between pre- and post-DecABT. The coloured bar represents *t*-statistics and coloured pixels in the brain represent peak values of significant voxels (*p*_unc_ < 0.001, for visualization). (*d*) Change in the number of trials towards gaming stimuli. The plot represents the change in the number of ‘*positive*’ trials, corresponding to the tendency towards gaming stimuli. A decrease in the number of trials represents a reduction of the tendency towards gaming stimuli. (*e*) Scatterplots between the change of the number of *positive* trials in gaming conditions and each participant’s peak value of changes in the precuneus. The scatterplot shows a correlation between the decrease in *positive* trials between pre- and post-Dec ABT and peak values of change in precuneus. The grey line represents the least squares fit.

Comparing pre- and post-brain activities based on AAT, there was significant deactivation within clusters centred in the cerebellum vermis (*p*_FDR_ = 0.006, *z* = 4.30, MNI coordinates: (−2 −66 −2), cluster size: 143), the right precuneus (*p*_FDR_ = 0.036, *z* = 4.39, MNI coordinates: (−12 −62 28), cluster size: 80) and the left cuneus (*p*_FDR_ = 0.036, *z* = 4.30, MNI coordinates: (12 −56 28), cluster size: 105) based on the contrast gaming avoidance > neutral avoidance (table 2 and figure 4*c*). There were, however, only qualitative changes in behavioural indexes related to trial-level attentional bias (figure 4*d*). In addition, there were no changes in the number of triggers across blocks during training (see electronic supplementary material, figure S11) and in the subjective questionnaire (see electronic supplementary material, figure S12). As a post-hoc explanatory analysis, we tested the correlation between the peak value of each ROI and changes in trial-level attentional bias. There was a strong relationship between improvement in attentional bias and precuneus reduction in activity to gaming approach (figure 4*e*; Spearman–Brown rho = 0.75, *p*_unc_ = 0.07). Moreover, though the effect was weaker than in the precuneus, the degree of correlation was moderate in the cuneus (Spearman–Brown rho = 0.61, *p*_unc_ = 0.17; electronic supplementary material, figure S13a). A similar relationship was not found with the cerebellar vermis (Spearman–Brown rho = 0.25, *p*_unc_ = 0.59; electronic supplementary material, figure S13b).

**Table 2.** Results of the comparison of pre- and post-brain activities based on AAT. Labels of each region are based on AAL3 [52].

conditions	region	peak				*p*_FDR_ values	cluster size
		*X*	*Y*	*Z*	*z*		
gaming avoidance versus neutral avoidance	Vermis_4_5	−2	−66	−2	4.30	0.006	143
	Precuneus_R	−12	−62	28	4.39	0.036	80
	Cuneus_L	12	−56	28	4.19	0.016	105

## 4. Discussion

In this study, we used MVPA of fMRI data captured during an approach–avoidance task to reveal the neural basis of temporal attentional bias towards game-related cues in gaming disorder. Our results indicate that the attentional bias state can be decoded from pre-stimulus neural activity in regions related to reward processing and attentional switching, particularly so in the bilateral insula. In addition, we conducted a proof-of-concept feasibility study of decoded attentional training for gaming disorder based on bilateral insula activity. We show preliminary results of qualitatively diminished attentional bias towards gaming stimuli and changes in brain activity in areas related to cue-reactivity and reward anticipation in behavioural addictions (precuneus and cerebellum).

MVPA classification of attentional bias state in the mesolimbic region, cerebellum, hippocampus and insula was significantly higher than chance levels across participants. However, results of control analyses thought to reflect general behavioural RT fluctuation based on neutral trials were also significant, with the notable exception of the insula. This similarity in decoding results across regions suggests that their activity patterns may not be necessarily related to gaming-specific attentional bias alone, but also to ordinary response time or general attention fluctuation. In contrast, the insular cortex underlies abnormal decision-making in addiction [65,66], and its pre-stimulus neural activity impacts perceptual and risk-taking valuation processes [67,68]. The insula has been suggested to regulate attention switching and working memory resources [69]. Moreover, previous work reported that deprivation from video gaming resulted in increased activation of the left insular activity when observing video gaming cues relative to neutral ones [49]. Though further research with a larger sample size is necessary to explore the interaction between types of gaming stimuli (personalised vs general) and conditions (gaming vs neutral), our findings could add to the evidence of temporal attentional bias state-switching for gaming depending on the insular activity in gaming disorder.

Beyond *a priori* regions of interest, our whole-brain search-ROI MVPA results found several regions in which accuracies were significantly higher than the chance level in attention bias decodability, of particular interest limbic and paralimbic cortices (consistent with ROI-based results), but also frontal and parietal lobes. Considering differences from control search-ROI results, these areas showed significantly higher decodability than the control condition, based on neutral trials. Since frontal regions track static attentional bias (calculated as an average signal across a whole session) in addiction [27] and action inhibition and preparation [70,71], frontal lobes may also relate to their interaction, i.e. the dynamics of attentional bias states. In addition, parietal regions appear to provide a priority map relating to preferential responses to previously rewarded stimuli [72]. Considering the possibility that value drives attention [73], the state of the priority map could be affected by activity fluctuations in this region, resulting in indirect changes to the temporal attentional bias state. Our results with control analyses, based only on neutral stimuli, suggest the specificity of regions related to addictive stimuli, i.e. gaming trials. Moreover, some brain regions obtained from whole-brain univariate analysis, e.g. the right angular gyrus, survived multiple comparison corrections in the search-ROI analysis. This overlap suggests that the brain regions involved in game-specific approach–avoidance movements are also related to attentional bias fluctuation based on endogenous neural activity.

Our preliminary investigation of DecABT indicates the possibility of brain activity modification even in the absence of clear changes in subjective gaming disorder assessment. This result is not necessarily surprising, given that previous DecNef studies have confirmed that even in the presence of physiological changes, changes in subjective awareness are not (immediately) apparent [57,74,75]. The task-based fMRI analysis showed reduced activation in the precuneus, cuneus and cerebellum vermis when pushing gaming images. The precuneus is important for cue reactivity in addiction, including behavioural addiction [76], and connectivity between this region and the insula is associated with craving in addiction [77]. Our results suggest that DecABT could change the neural substrate associated with the insula, which is essential for reactivity to addictive stimuli. Moreover, activity centred in the cerebellum vermis was also reduced (though displaying no clear relationship with behavioural changes). Recent studies suggest that the cerebellum is significantly involved in gaming disorder [35]. In particular, because the vermis is reportedly essential for reward anticipation [78], reduced activity when avoiding gaming stimuli suggests that the training could lead participants to control their reward processing. However, the stronger correlation between the peak of changes in precuneus activity and behavioural changes could indicate the importance of the precuneus as a marker of training effects. Further work is definitely needed to answer these questions, as the current sample size in the proof-of-concept manipulation was small and precludes conclusive arguments.

This novel method provides several advantages compared to previous interventions using (decoded) neurofeedback. First, although traditional neurofeedback uses external rewards, i.e. money, to reinforce specific brain states connected to the target behaviour [79], DecABT provides just-in-time intervention to change the behaviour. Considering the impracticality of using external rewards in actual clinical applications, developing protocols that can lead to brain and behaviour change without relying on external rewards is essential. Second, our approach reduces the burden on participants compared to past attempts. In previous studies that attempted to train sustained attention and change attentional bias in depression [80,81], participants had to continue manipulating their brain states to maintain appropriate attentional states during the experiment. In DecABT, it is not necessary to directly control one’s brain states, and participants only need to respond to tasks (trials, problems, questions, etc.) that appear at a specific time. Thus, the re-association between brain activity and a specific behavioural response may occur without participant awareness. Reducing participant burden is essential to sustain motivation and prevent drop-out, especially in addiction disorders [82].

There are several considerations to our study. In this study, we used personalised stimuli, meaning that picture features were not controlled across participants in terms of valence or low-level visual information. While future studies should consider controlling stimuli of specific addictive games, the present results indicate that temporal attentional bias state is associated with specific brain regions common across subjects, even if the title and type of game differ. The method to detect temporal attentional bias should also be considered. Although we used an established baseline based on neutral averaged RT [40] and trial-level attentional biases, which had strong within-participant reliability, this may include confounders such as ordinary response time fluctuation and order effects. Moreover, the MVPA performance differed significantly by direction (approach/avoidance) in some ROIs and conditions. This result suggests attentional bias states may be influenced by specific factors, in particular, related to the type of avoidance response, i.e. pushing. Since the pulling and pushing responses have different accompanying characteristics, calculating a single value from the reaction times of separate actions might be problematic [83]. Future studies should consider different methods to detect attentional bias states, such as using a running average of neutral RT or modelling approaches [84,85]. In addition, both MVPA and DecABT had relatively small sample sizes, limiting the strength of inference. Because we did not include potential control conditions in the DecABT, it is difficult to definitely rule out other possible influences, such as pseudo-learning effects from repeated exposure to gaming stimuli. However, our triggering design based on decoded brain activity patterns should decrease such confounding effects because it is a prospective prediction (predicting future recurrence probability of attention bias towards gaming). Finally, related to the DecABT training, self-reported symptoms did not change immediately after the experiment. It is possible that modulation of attentional bias could sequentially ameliorate symptoms later, i.e. on a scale of weeks or months [23]. Future studies, informed by our preliminary findings, should follow-up participants over multiple time points post-training.

In summary, we found that the attentional bias state in gaming disorder can be inferred from multivoxel patterns of insular endogenous activity. While other brain areas also track attentional states, the insula appears to be doing so specifically in relation to gaming-related cues. Moreover, although this is a preliminary pilot test with several limitations, ABM using a form of real-time decoding of brain activity patterns (DecABT) suggests that it could be an effective intervention for gaming disorder. This method could provide just-in-time training based on neural states reflecting a participant’s ongoing level of attentional bias. More importantly, at this stage, it can also help to understand better the causal relationship between attentional bias, its variability in time, and symptoms. This approach may allow novel experimental design as well as tailor-made treatment of gaming disorder, other addiction disorders and broader attention deficit disorders.

## Data Availability

The data supporting this study’s findings and custom codes used to generate results and figures are available at [86]. Supplementary material is available online [87].
